# Drug development in LMICs: could the emerging Indian model usher the southeast Asian region?

**DOI:** 10.1016/j.lansea.2024.100464

**Published:** 2024-08-16

**Authors:** Bharat Pant, Jayant Goda, Vikram Gota

**Affiliations:** a Department of Clinical Pharmacology, Advanced Centre for Treatment Research & Education in Cancer (ACTREC), Tata Memorial Centre, Kharghar, Navi Mumbai, Maharashtra, 410210, India; b Homi Bhabha National Institute, Anushakti Nagar, Mumbai, Maharashtra, 400094, India; c Department of Radiation Oncology, Advanced Centre for Treatment Research & Education in Cancer (ACTREC), Tata Memorial Centre, Kharghar, Navi Mumbai, Maharashtra, 410210, India

**Keywords:** Oncotherapeutics, LMIC, Drug development, Industry-academia partnership, Chlorophyllin, Diselenodipropionic acid, DSePA, Novel asparaginase, 6-MP PFOS, 6-mercaptopurine powder for oral solution, Drug development India, Drug development LMIC, Drug development southeast Asia, CAR-T, NexCAR19

## Abstract

Low-income and middle-income countries (LMICs) of southeast Asia are passing through a similar phase as India in their tryst with the development of novel drugs. They are beginning to break away from their dependency on the institutions of our developed world. Over the past few years, Tata Memorial Centre—India's premier cancer centre—has shown the tenacity to develop drugs within the national frontiers. By collaborating with the domestic pharmaceutical industries, it has been able to have a steady pipeline of drugs under development, with two of them receiving marketing authorization recently. Lately, Indonesia and Vietnam have also shown an inclination towards public-private partnerships for similar motives. However, due to prolonged innovative stagnation, the entire drug development machinery faces challenges stretching all the way from arranging funds to persuading regulatory bodies. In this Viewpoint, we have tried to address a few of those issues and their potential solutions, with the intention to share our own experience which might be useful to other LMICs in connecting some adamant dots.

After almost a decade of the “Make-in-India” initiative, India has surely exuded the potential of being self-reliant in manufacturing a range of products, from toys to semiconductors. The development of indigenous technologies is crucial to ensure self-sustainability in the future. In the healthcare sector, the government has taken initiatives to promote home-grown medical devices, like stents and prostheses.^1^ Although, India possesses a thriving market of generics and biosimilars, to this date, India is dependent on the high-income countries (HICs) in western part of the globe for drug discovery and development. The country had shown an initial spark in this direction more than a century ago, when Urea Stibamine was developed for the treatment of Kala Azar in the School of Tropical Medicine, Calcutta in 1921.^2^ Ever since, barring a few flickers of development of a handful of drugs,^3^^,^^4^ the journey is largely dormant, compared to the progress India has shown in other areas.

Drug research in the HICs in the western part of the globe has hardly ever been driven by the unmet needs of low-income and middle-income countries (LMICs). It is unreasonable to expect the opposite. Not surprisingly, it took an epidemic for the global health community to focus on developing a vaccine for Ebola when that could have been possible years ago.^5^ In India, events in the recent past have indicated that perhaps even the patent laws are not entirely in line with the ideologies that seem to drive the business models of industries, which means we are yet to strike a balance between profitability from the drug development business and the societal good.^6^ However, there exists an alternative argument that lauds Indian patent laws for protecting against evergreening. Incidentally, some experts have recommended that countries like Cambodia should draw inspiration from India while formulating their patent laws. It is pertinent to note that Cambodia is presently classified as a Least Developed Country (LDC) by the UN and is yet to incorporate public health-related Trade-Related Aspects of Intellectual Property Rights (TRIPS) flexibilities into legislation. But, as it transitions from this status in 2033, it would be required to enforce patent laws.^7^ On the other hand, Indonesia, another Southeast Asian LMIC, is often mentioned for its lack of flexibility in the Intellectual Property Rights framework, that has proven to be detrimental to innovation.^8^

In the context of cancer therapeutics, patients from LMICs constitute a mere 10–20% of participation in clinical trials which does not justify the 70–80% share of these countries in the global burden. Notably, a majority of the contemporary trials are on expensive agents that are largely unaffordable in the LMICs.^9^ The common cancers prevalent in LMICs are often overlooked when it comes to research.^10^ For example, gastric adenocarcinoma ranks third in terms of mortality caused globally by malignancies, with its major share in the LMICs, yet the majority of vaccines against *Helicobacter pylori* infection are only in the early phases of research.^11^ Despite advancements in screening and vaccination, cervical cancer continues to burden the LMICs. Upon advancing locally, its prognosis plummets sharply, with high rates of metastasis and recurrence, and a 5-year overall survival of 50–60%.^12^ But, the efforts to find an efficient treatment for cervical cancer do not justify the huge burden of the disease. Likewise, oral cancer plagues southern and southeast Asia, but the treatment options remain limited. This marks a great opportunity for the domestic pharmaceutical industries in the LMICs to step in and develop drugs for these indications which could be financially rewarding and morally gratifying.

Progress in the Indian pharmaceutical sector and the research institutes have long been prompting industry-academia collaboration as a solution to this issue. Industries possess the resources, technology, and equipment but are generally risk-averse to invest heavily in research and development. Academic institutes harbour the intellect, the know-how, and the appetite for drug development, but are undone for want of funds. This augurs well for a marriage between the two where the industry can derisk capital investment in hiring manpower and setting up labs by funnelling funds to preferred academic partners. Such collaborations have been effective in the western part of the globe, where industry-academia collaborations have produced new molecules (Fig. 1). Two such historical examples of drugs that came out from the USA in the 1950s are Cortisone and Streptomycin, thus demonstrating a successful partnership between the two.^13^

**Fig. 1:**
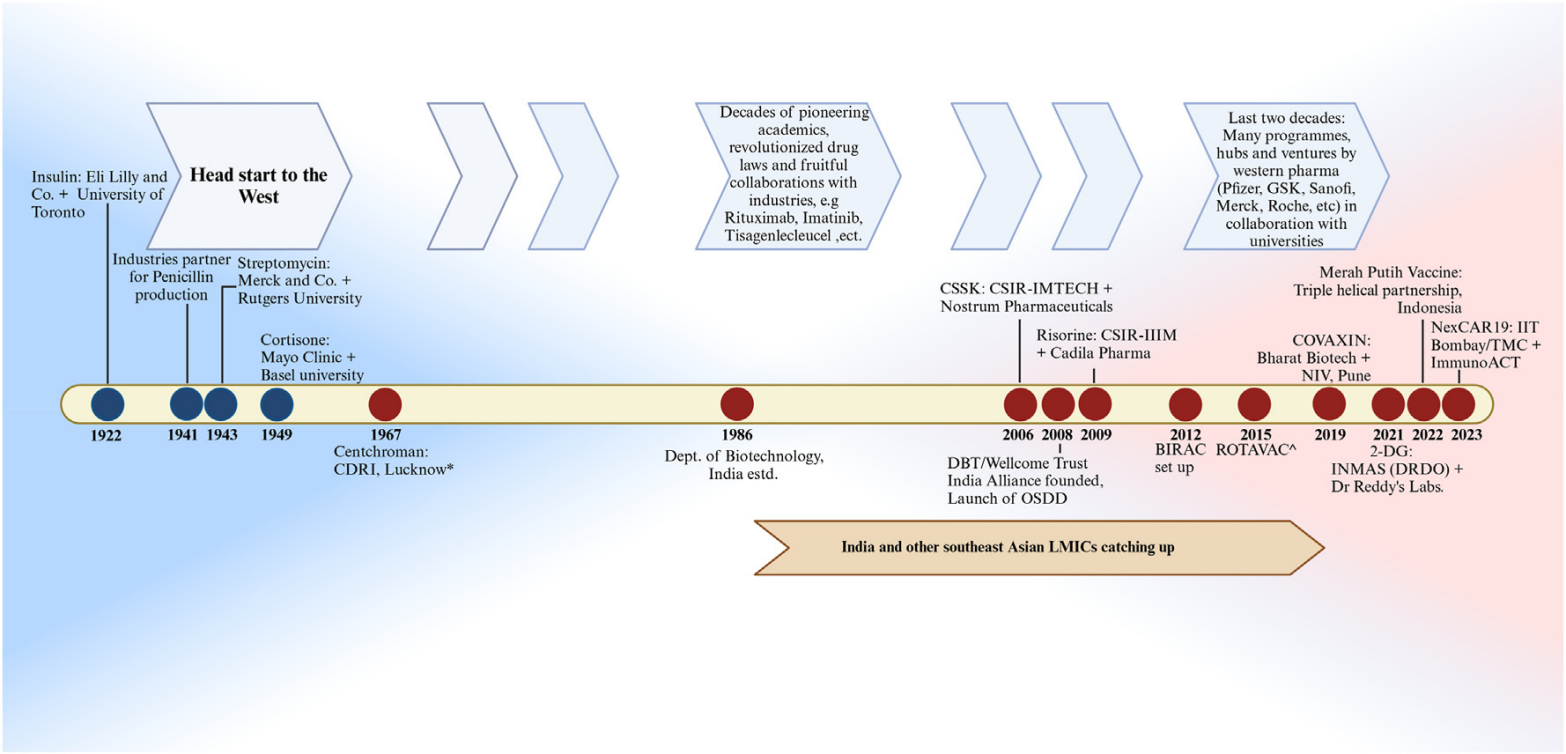
**Timeline of significant events (in red) in the history of drug development by pharmaceutical industry-academia collaborations in India, in comparison to much early start by the counterparts in the western part of the globe (in blue)**. ∗After being developed in 1967, Centchroman was manufactured in early 1990s by Torrent Pharmaceuticals and later, Hindustan Latex Limited Lifecare; ˆROTAVAC was developed by 25 years of collaboration of Ministry of Science and Technology, US Government institutes, various Indian government institutes and NGOs, Bill and Melinda Gates Foundation and Bharat Biotech India Limited. Abbreviations: CDRI, Central Drug Research Institute; CSSK, Clot-specific Streptokinase; DBT, Department of Biotechnology; OSDD, Open Source Drug Discovery; BIRAC, Biotechnology Industry Research Assistance Council; NIV, National Institute of Virology; 2-DG, 2-deoxy-d-glucose; INMAS, Institute of Nuclear Medicine and Allied Sciences; DRDO, Defence Research and Development Organisation; TMC, Tata Memorial Centre. (Figure not to scale).

In India, Centchroman was a benchmark contraceptive pill developed by the Central Drug Research Institute (CDRI), Lucknow, and manufactured by Torrent Pharmaceuticals, Ahmedabad, and later by Hindustan Latex Limited (HLL) Lifecare, Thiruvananthapuram under.^14^ The CSIR-IMTECH (Institute of Microbial Technology), Chandigarh collaborated with Nostrum Pharmaceuticals of the United States to develop the Clot Specific Streptokinase (CSSK).^15^ CSIR-IIIM (Indian Institute of Integrative Medicine), Jammu, and Cadila Pharma joined forces to produce Risorine, an anti-tubercular combination.^3^^,^^16^ ROTAVAC, India's first indigenously developed Rotavirus vaccine, which was launched in 2015, was the result of over 25 years of innovative collaboration amongst Government of India's Ministry of Science and Technology, various institutes of the Indian government, institutes in the USA like the National Institute of Health, Gates Foundation, and the Indian company Bharat Biotech India Limited.^17^ The National Institute of Virology, Pune, and Bharat Biotech came together to bestow us with India's first indigenous vaccine against SARS-CoV-2, COVAXIN.^18^ Another adjunctive therapy targeting the COVID-19, which was approved for emergency use by the Drugs Controller General of India was the powder formulation of 2-deoxy-d-glucose (2-DG). It was developed by the Institute of Nuclear Medicine and Allied Sciences (INMAS) under the Defence Research and Development Organisation (DRDO), in collaboration with Dr Reddy's Laboratories. Notwithstanding the uncertainty surrounding its efficacy, this was a noteworthy effort by the emerging Indian research ecosystem to fight the epidemic.^19^

The Tata Memorial Centre—India's premier cancer centre—has been developing such partnerships with industries, and our experience so far has been quite fruitful. Recently, we witnessed the launch of India's first home-grown anti-cancer CAR-T cell therapy, actalycabtagene autoleucel (NexCAR19), which was the product of a successful Industry-Academia collaboration between the Indian Institute of Technology, Bombay, and Tata Memorial Centre, along with the industry partner, ImmunoACT. By reducing cost of infusion from around US$ 400,000 to US$ 40,000 through rigorous R&D efforts and collaboration with several cancer centers across India, the therapy would help in increasing affordability and accessibility to people in LMICs.^20^ Through different types of collaborative models, we have been able to successfully develop four drugs so far. Broadly, the nature of our collaboration with industry partners could be divided into the following two categories.

The first model of industry-academia partnership set the platform for the development of three drugs, namely diselenodipropionic acid (DSePA), chlorophyllin, and novel asparaginase mutants for the treatment of acute lymphoblastic leukaemia (ALL). DSePA is an organoselenide that has exhibited dual chemo-preventive and chemo-therapeutic, as well as radioprotective activity.^21^^,^^22^ l-asparaginase, used in the treatment of paediatric ALL, has several limitations in the form of immunogenicity, antigenicity, short half-life, accompanying glutaminase activity, and toxicities thereof. In collaboration with Indian Institute of Technology, Indore, two novel variants of l-asparaginase were constructed using protein engineering approach that demonstrated better pharmacological properties as compared to the native asparaginase.^23^ Essentially, the intent was to leverage the excellent scientific acumen for drug discovery and development existing in large academic institutions, followed by the transfer of technology to pharmaceutical industries for further development in the later stages.

This model could be further divided into two sub-categories based on the source of funding. In the case of DSePA, l-asparaginase, funding was obtained from a federal agency. However, the funding mechanism for chlorophyllin, a semi-synthetic derivative of chlorophyll that has shown radio–protective activity in a phase 2 trial of radiotherapy-induced haemorrhagic cystitis (NCT05348239) was a bit different.^24^ The research centre that developed the drug entirely supported its development from bench to clinics, which in this case was, BARC (Bhabha Atomic Research Centre).

The second strategy of collaboration reflects a close-knit arrangement, where instead of contributing sequentially, the industry and academia work together right from the beginning, wherein the industry may entirely commit to drug development and conducting clinical trials, while the academic centre may act as an advisor to guide through each step, a model used for the development of the liquid formulation of 6-MP (6-mercaptopurine). To make this plan come forth, the start-up company was constantly guided at every step by incisive directions from oncologists and clinical pharmacologists in a range of domains including nature of the formulation, clinical development plan, as well as use of pharmacokinetic modelling and simulation for dosing recommendations.^25^^,^^26^

Owing to the advantage the HICs in the western part of the globe possess in terms of the added years of such collaborations, they have more diverse models that LMICs may draw inspiration from, e.g. Johnson & Johnson, a USA-based pharmaceutical giant, finances a company called Nucleome Therapeutics Limited that has emerged out of and is based on the research conducted at the Oxford university and frequently collaborates with its researchers. Johnson & Johnson also hosts four innovation hubs and 11 laboratories across the world, that provide one-stop access for the scientists, entrepreneurs, and emerging companies to the science and technology experts, with a scope of collaborations, as well as identification and development of innovations to fulfil the unmet needs of people.^27^ Some Indian pharmaceutical companies too have begun to invest in building research foundations, a public-private partnership model to fuel drug discovery.^3^ International pharma giants like Pfizer have already begun to expand their research and development bases to India.^28^ The Indian Council of Medical Research (ICMR) has also come up with an initiative to develop Phase 1 clinical trial centres of excellence across the country, equipped with adequate infrastructure and suitably trained manpower. Any company or research institute having obtained regulatory approval to conduct early phase clinical trials of their molecules may utilise these facilities, with possible financial support from ICMR to conduct these trials.^29^

The Council of Scientific & Industrial Research (CSIR), India seems to acknowledge the importance of collaborations. In 2008, it launched Open Source Drug Discovery (OSDD), a consortium that provided a global platform for scientists, doctors, and students to collaborate and find solutions to complex problems associated with discovering novel therapies for neglected tropical diseases like tuberculosis and malaria.^30^ Within 7–8 years of its introduction, it had reportedly partnered with several international organisations and gained more than 9000 participants from 130 countries. The volunteers re-annotated *Mycobacterium tuberculosis*, to propose novel targets and potential therapy, by spending only a fraction of (0.5%) what a pharmaceutical industry would exhaust to develop a new drug. Although the programme faced funding issues and was eventually absorbed into the India TB Research and Development Consortium, it was pioneering, and influential, that laid the early seed for the culture of drug discovery in India. We feel that there is no reason why such similar platforms cannot be used to develop therapies for common cancers in LMICs.

However, the collaborative atmosphere required for innovation is yet to be established in the country. India's leading biopharmaceutical company, Biocon partnered transnationally with Cuba's Molecular Immunology Centre to further develop and manufacture their then-novel drug for psoriasis, Itolizumab.^31^ Such North-South collaborations are a sought-after arrangement in recent times, that attempts to bail out a developing country of its issues in the health sector. Other LMICs from the southeast Asian region also often look forward to such collaborations which, over the years, have addressed various issues spanning from developing affordable treatments^32^ and vaccines^33^ for neglected tropical diseases to exploring traditional regional medicine for cancer therapeutics.^34^

International collaborations are undeniably welcome, but India houses a plethora of biotechnology-based research institutes, both public as well as private, and ranks high globally in research in chemistry and basic life sciences. In essence, we must also learn to water our own turf to sprout home-grown molecules for our industries to further work upon and develop. Interestingly, firms from HICs in the western part of the globe have started eyeing collaborations with the local investigators of the LMICs, and talks are already around in the town about identifying and solving the hurdles that exist in their way.^10^ Undoubtedly, this would be yet another virtuous attempt to consolidate that global health is everybody's responsibility. But the contention of us finally taking our charge still stays and such initiatives by the western part of the globe might as well be taken as an inspiration to solve our problems.

The story is not all that different for the rest of the LMICs across southeast Asia, where except for a few sporadic partnerships in recent years, the industries have mostly stayed distant from academia. Only 1.3% of the scholarly output from the south Asian region is attributed to such partnerships.^35^ Recently, there was a triple-helical partnership between Vietnam-based Boston Pharma, the School of Medicine, and the Institute for Drug Quality Control, for training, research, and development of pharmaceutical products.^36^ Known for having the lowest R&D budget amongst the G20 nations, Indonesia also turned towards such a partnership for the development of their home-grown ‘Merah Putih vaccine’ for SARS-CoV-2.^37^ Various experts have suggested industry-academia collaborations as a means to deal with its limited resources and infrastructure.^38^ Established in 2013, DA-EWG (Drug Discovery Alliances-Expert Working Group) is an industry-driven open innovation platform, consisting of nine Asian member countries, with the overall goal to develop innovative medicines from Asia. Within this platform, it was reported that the last five years have witnessed the collaboration of two Japanese companies with government and academic institutes of Thailand, yet another fellow LMIC. As reported by the group's leader, Dr Megumi Ikemori at the 13th Asia Partnership Conference of Pharmaceutical Associations (APAC) in April 2024, this collaboration allowed the sharing of know-how regarding drug discovery, in addition to training of young researchers.^39^ An outline of prospective solutions offered by Industry-Academia collaborations for existing obstacles to drug research is represented in Table 1.

**Table 1:** Industry-academia collaborations as a means to resolve issues in drug development in India and other southeast Asian low-income and middle-income countries.

Problem	Solution	Examples
Lack of infrastructure in academia to continue drug development at later stages	Technology transfer to industry	CSSK (CSIR-IMTECH and Nostrum Pharmaceuticals) DSePA (TMC, BARC, and CLINEXEL Life Sciences) Novel asparagenase variants (TMC, IIT Indore, and DK Biopharma Pvt. Ltd.) Chlorophyllin (BARC, TMC and IDRS Labs, Bangalore) Risorine (CSIR-IIIM & Cadila Pharma) COVAXIN (National Institute of Virology and Bharat Biotech) NexCAR19 (IIT Bombay-TMC and ImmunoACT) Oral 2-deoxy-d-glucose powder for SARS-CoV-2 (INMAS-DRDO and Dr Reddy's Laboratories) ROTAVAC^a^ Centchroman (CDRI, Lucknow and Torrent Pharmaceuticals, Ahmedabad–HLL Lifecare, Thiruvananthapuram)
Industries may lack direction to go forward in development of any drug	Academia guides industries	6-MP PFOS (TMC and IDRS Labs)
Lack of innovative mind-set in the industry	Industries developing their own research centres	Research foundations by companies like Dr. Reddy's Laboratories and Ranbaxy Laboratories
Lack of communication between experts or enthusiasts	Online consortium for global interdisciplinary collaboration	Open Source Drug Discovery (OSDD)
Lack of trained researchers and infrastructure	Government interventions and collaborations to lay the framework for training, and research in drug development	National Testing Agency and Grants for proposals by the Department of Biotechnology (DBT), India 3-party collaboration amongst the School of Medicine—Vietnam National University, Institute for Drug Quality Control (IDQC), and the Boston Pharma, Vietnam

a ROTAVAC: innovative public-private collaboration amongst Government of India's Ministry of Science and Technology, US Government institutes like National Institute of Health, government institutes and NGOs in India, Bill and Melinda Gates Foundation, Bharat Biotech India Limited. Abbreviations: CSSK, Clot-specific Streptokinase; CSIR, Council of Scientific & Industrial Research; IMTECH, Institute of Microbial Technology; DSePA, diselenodipropionic acid; TMC, Tata Memorial Centre; IIT, Indian Institute of Technology; INMAS, Institute of Nuclear Medicine and Allied Sciences; DRDO, Defence Research and Development Organisation; CDRI, Central Drug Research Institute; HLL, Hindustan Latex Limited; BARC, Bhabha Atomic Research Centre; IIIM, Indian Institute of Integrative Medicine; 6-MP PFOS, 6-mercaptopurine powder for oral suspension.

So, what could be the reason for such a paucity of indigenously developed molecules that could have sprouted by industry-academia collaborations? From our experience, funding could be one of the potential roadblocks to drug development that the stakeholders might face. As discussed above, federal grants from various agencies are a way out of this. We used the scheme from BIRAC (Biotechnology Industry Research Assistance Council) to aid the development of two of our drugs, l-asparaginase and DSePA. Apart from BIRAC, the Department of Biotechnology, Government of India also lays on another such initiative named the ‘Biotechnology Industry Partnership Programme’ (BIPP). Other schemes in this direction include NMITLI (New Millennium Indian Technology Leadership Initiative) and OSDD (Open Source Drug Discovery) by the Council of Scientific & Industrial Research (CSIR), New Delhi.^3^ The BIRAC, DBT, and government of India have envisioned India as a global leader in biotechnology-based drug research. Through their policies, they have made efforts that focus on industry-academia collaborations and promoting entrepreneurship, to fund various biotechnology-based start-ups, while also supporting research institutes as well as Small and Medium-scale enterprises. Introducing such schemes is reassuring and indicative of the government's efforts to deal with the bottlenecks.

In India, except for a few institutes, like the Indian Institute of Technology Bombay, most of the institutes also seem to lack a revenue-sharing model that could ensure an equitable distribution of risks and rewards amongst the collaborating partners. There is a need for other research institutes to adopt similar models. Lack of awareness among the faculty from various universities regarding such grants and schemes coupled with exhaustive academic and administrative work prevent them from exploring such opportunities. Secondly, the curriculum of advanced training programs does not necessarily favor innovation. The curriculum for the course of MD Pharmacology, for example, was recently updated by the National Medical Council (NMC) of India to inculcate internships in the pharmaceutical industry, which is a step in the right direction. Workshops and short-term training programs could be conducted to address the issue of lack of awareness regarding patents and intellectual property rights among academic researchers.^40^ Unwillingness among academicians and industry personnel to collaborate and transfer knowledge might often be observed.^41^ Lack of communication between scientists, clinicians, and industry has been a historical barrier to drug development. The development of chlorophyllin as a radioprotector and the powder for oral suspension of 6-mercaptopurine for ALL clearly demonstrated how this barrier could be overcome for a successful translation from bench to bedside.

As it turns out, the complex and lengthy regulatory processes is a necessary evil, but one could be hopeful for the evolution of an approval process that is both transparent and efficient. The New Drug and Clinical Trial Rules (2019) are a good effort in this direction, establishing clear definitions and delegation of powers. The Central Licensing Authority, now, has to respond within 30 working days of application for conducting a clinical trial of a new drug or investigational new drug as part of discovery, research, and manufacture in the country, beyond which there is a provision of deemed approval. Additionally, there exists a clause wherein the sponsor or applicant may apply for expedited review for approval of a new drug that's intended to treat a life-threatening or rare disease, is orphan, provides a significant advantage in terms of safety and efficacy, or is developed for disaster use in an extraordinary situation. Such policy changes have led to shorter approval timelines for new drug clinical trials, thus allowing a smooth and unhindered drug development process.^42^

Such developments could set a precedent for LMICs in southeast Asia like Indonesia, which is reported to have strict regulations for pharmaceutical industries, further imbalanced by limited research facilities, leading to unnecessary delays in getting the drug to the market. This is accompanied by an additional challenge to lower drug prices to provide Universal Health Coverage (UHC), which further lessens a company's incentives for venturing into drug development.^38^ Even Vietnam is known for delays in regulatory approval of new drugs, where a single administrative branch of its Ministry of Health is in charge of everything from approving and managing market authorisation to licensing.^43^ But the scenario is expected to improve by the implementation of its “National Strategy for Development of Vietnam's Pharmaceutical Industry to 2030”, which has the notion of improving the performance of the regulatory systems as an integral part of its framework.^44^

Malaysia, on the other hand, already boasts shorter regulatory and ethics approval timelines, compared to the likes of Japan, Singapore, and Taiwan. Like Vietnam, Laos, and Cambodia, Malaysia is yet to capitalise on the generic drug market, let alone the notion of indigenous drug development, but over the years, the scenario of clinical trials has been vastly improved by Clinical Research Malaysia (CRM), a non-profit entity established by its Ministry of Health. A Phase 1 Realization Project has also been commenced to mature its early phase research competency.^45^

Unlike most other countries, the regulatory framework in India has been expanded to include phytopharmaceuticals and not just synthetic compounds. Even Vietnam, in its National Strategy (discussed above), has emphasised developing medicinal herbs.^44^ Natural products drug discovery is also one of the pillars of the DA-EWG platform, of which, Thailand, India, and Malaysia are also members.^46^ Notably, the Thailand Board of Investment also grants corporate tax incentives to the pharmaceutical companies to produce conventional and traditional medicines.^47^

Drug development is fuel for the field of medical healthcare, and innovations in this sector are important if LMICs hope to find their own solutions to their indigenous problems. In all these years of dependency upon the HICs in this area, we have consequently witnessed outrageous costs of medications. The fact that the market share of the Indian brands of cardiac stents grew from 30% to 55% within three years of the National Pharmaceutical Pricing Authority (NPPA) imposing a cap on their pricing, is a testament to the notion that local research and development can not only decrease the monetary burden of healthcare expenditure but also simultaneously boost the local industries.^48^ Based on our experience and vision, we envisage a system that inculcates the mindset to innovate and provides the infrastructure to do so (Fig. 2). Federal grants are a solution to the monetary issues and lack of resources, but the process of disbursement may have to be streamlined. The granting schemes and mechanisms should be adequately publicised to have a wider reach. Over the years, other LMICs from the southeast Asian region have also started to host research grants to promote drug research and development, e.g. research grants from Badan Riset dan Inovasi Nasional (BRIN, Indonesia), PCHRD (Philippine Council for Health Research and Development) Research Grant, and Enterprise Support Program by National Foundation for Science & Technology Development (Vietnam), to name a few (Table 2).

**Fig. 2:**
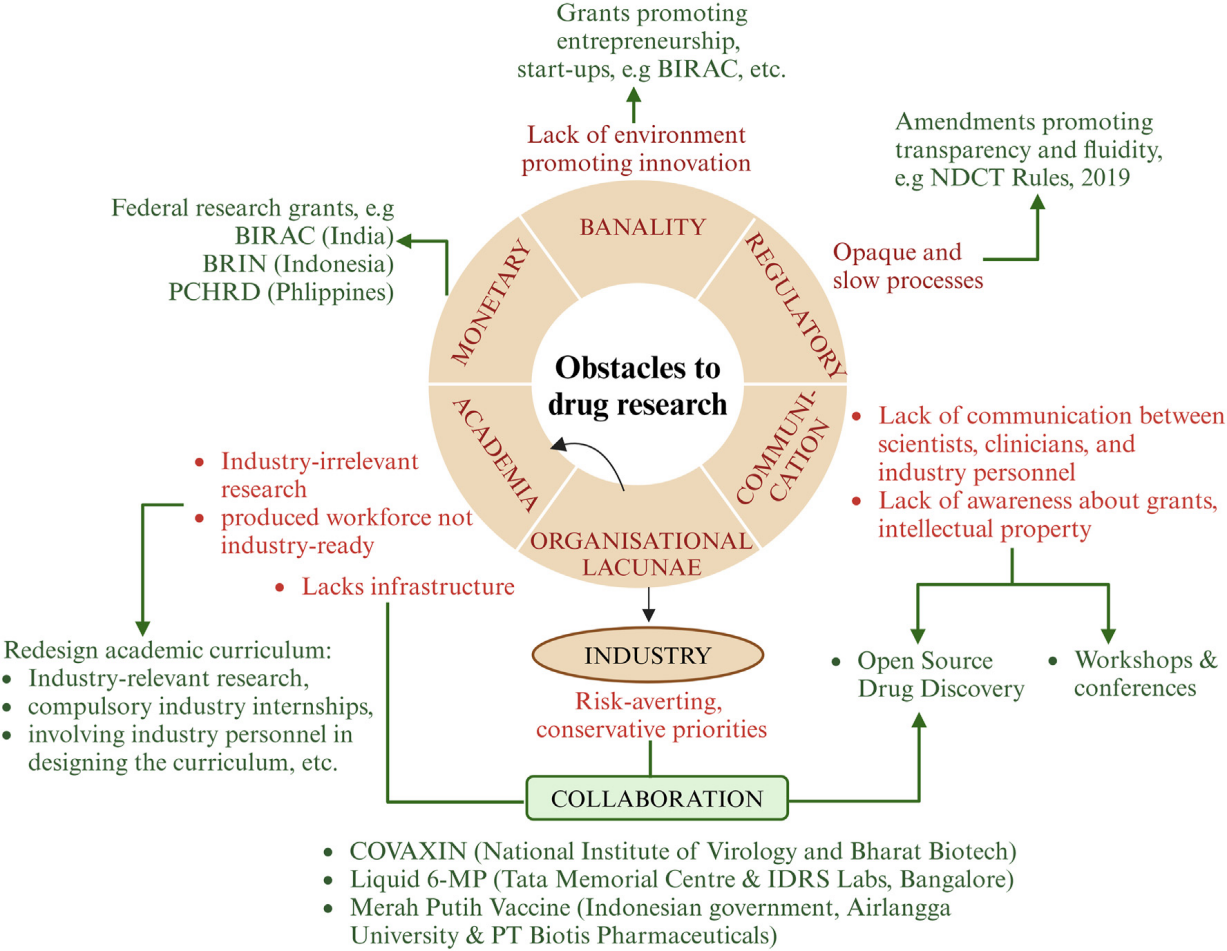
**Obstacles (in red) to drug research in India and other southeast Asian LMICs and their potential solutions (in green)**. Abbreviations: BIRAC, Biotechnology Industry Research Assistance Council; BRIN, Badan Riset dan Inovasi Nasional; PCHRD, Philippine Council for Health Research and Development; NDCT, New Drug and Clinical Trial; 6-MP, 6-mercaptopurine.

**Table 2:** Funding agencies bridging gaps in drug development in India and other southeast Asian low-income and middle-income countries.

Purpose	Agency
Capacity building: Postgraduate programmes and fellowships	DBT, India BRIN, Indonesia PCHRD, Philippines Ratchadapisek Research Funds, Faculty of Medicine, Chulalongkorn University, Thailand
Industry-academia interface to de-risk biotechnological research and promote technology transfer	BIRAC DBT/Wellcome Trust India Alliance
Promote interdisciplinary research	CSIR DBT/Wellcome Trust India Alliance
Monitor progress of funded research and decide correction/renewal	CSIR
Ease of submission of research proposals: Principle Investigator may submit directly Online submission and monitoring	CSIR e-PMS (electronic Project Management System) by ICMR
Financial support for research on Ayurvedic medicines	Ministry of AYUSH
Supporting startups	DBT Indonesian Research and Innovation Fund by BRIN Applied Innovation Fund, Technology Development Fund, Strategic Research Fund by MOSTI, Malaysia
Broader motive of supporting investigators	MOH Research Grant, Malaysia Health Systems Research Institute, Thailand

Abbreviations: DBT, Department of Biotechnology; BRIN, Badan Riset dan Inovasi Nasional; PCHRD, Philippine Council for Health Research and Development; BIRAC, Biotechnology Industry Research Assistance Council; CSIR, Council of Scientific & Industrial Research; ICMR, Indian Council of Medical Research; AYUSH, Ayurveda, Yoga and Naturopathy, Unani, Siddha, and Homeopathy; MOSTI, Ministry of Science, Technology and Innovation; MOH, Ministry of Health.

We advocate an environment that boosts collaborations between academia and industry. Due to our geographical, historical, diplomatic, and economic proximity, a similar framework could also be adopted by other LMICs of the region, with minor adjustments as per their local beliefs, cultures, and environment.

## Contributors

Based on their research and development experience with DSePA, novel asparagenase variants, chlorophyllin, and liquid formulation of 6-mercaptopurine, VG and JG conceptualised and designed the framework for the manuscript. BP carried out the literature search to acquire and curate the information and prepared the original draft of the manuscript. BP also put together the figures and tables under the supervision of VG and JG. VG and JG reviewed and edited the manuscript. All the authors assessed and verified the underlying data and approved the final manuscript.

## Declaration of interests

We declare no competing interests.
